# TRAIL-Deficiency Accelerates Vascular Calcification in Atherosclerosis via Modulation of RANKL

**DOI:** 10.1371/journal.pone.0074211

**Published:** 2013-09-05

**Authors:** Belinda A. Di Bartolo, Siân P. Cartland, Hanis H. Harith, Yuri V. Bobryshev, Michael Schoppet, Mary M. Kavurma

**Affiliations:** 1 Centre for Vascular Research, University of New South Wales, Sydney, NSW, Australia; 2 School of Medical Sciences, University of New South Wales, Sydney, NSW, Australia; 3 Department of Biomedical Sciences, Faculty of Medicine and Health Sciences, Universiti Putra Malaysia, Serdang, Malaysia; 4 Department of Internal Medicine and Cardiology, Philips University, Marburg, Germany; Harvard Medical School, United States of America

## Abstract

The osteoprotegerin (OPG) and receptor activator of nuclear factor-κB ligand (RANKL) cytokine system, not only controls bone homeostasis, but has been implicated in regulating vascular calcification. TNF–related apoptosis-inducing ligand (TRAIL) is a second ligand for OPG, and although its effect in vascular calcification *in vitro* is controversial, its role *in vivo* is not yet established. This study aimed to investigate the role of TRAIL in vascular calcification *in vitro* using vascular smooth muscle cells (VSMCs) isolated from TRAIL^−/−^ and wild-type mice, as well as *in vivo*, in advanced atherosclerotic lesions of TRAIL^−/−^ApoE^−/−^ mice. The involvement of OPG and RANKL in this process was also examined. TRAIL dose-dependently inhibited calcium-induced calcification of human VSMCs, while TRAIL^−/−^ VSMCs demonstrated accelerated calcification induced by multiple concentrations of calcium compared to wild-type cells. Consistent with this, RANKL mRNA was significantly elevated with 24 h calcium treatment, while OPG and TRAIL expression in human VSMCs was inhibited. Brachiocephalic arteries from TRAIL^−/−^ApoE^−/−^ and ApoE^−/−^ mice fed a high fat diet for 12 w demonstrated increased chondrocyte-like cells in atherosclerotic plaque, as well as increased aortic collagen II mRNA expression in TRAIL^−/−^ApoE^−/−^ mice, with significant increases in calcification observed at 20 w. TRAIL^−/−^ApoE^−/−^ aortas also had significantly elevated RANKL, BMP-2, IL-1β, and PPAR-γ expression at 12 w. Our data provides the first evidence that TRAIL deficiency results in accelerated cartilaginous metaplasia and calcification in atherosclerosis, and that TRAIL plays an important role in the regulation of RANKL and inflammatory markers mediating bone turn over in the vasculature.

## Introduction

Atherosclerosis, a chronic arterial disease that affects the entire artery tree can lead to myocardial infarction, stroke and gangrene, and is the most common cause of mortality worldwide. Atherosclerosis is associated with extracellular calcium and calcium accumulation in the plaque. In fact, calcified tissue represents 15-20% of total plaque area with vascular calcification being increasingly recognised as a risk factor for cardiovascular disease, suggesting that calcium may be a marker of atherosclerosis [1].

Studies in the last decade suggest that calcification in the vessel wall is an active process, regulated in a manner analogous to orthotopic bone formation [2]. Osteochondrogenic cells have been identified in calcified vascular tissue [3], [4], [5], [6], [7] and it is suggested that the triad cytokine system of osteoprotegerin (OPG), receptor activator of nuclear factor-κB ligand (RANKL) and its receptor RANK, may act as a link between vascular calcification and bone metabolism [8]. RANKL binds its cellular receptor RANK expressed on osteoclasts to initiate osteoclastogenesis [8], [9]. RANKL can also bind its soluble decoy receptor, OPG. Binding of RANKL to OPG inhibits differentiation, maturation, activity and survival of osteoclasts, subsequently inhibiting mineralisation [10].

While the role of OPG and RANKL in bone metabolism is clear, their role in vascular calcification is still elusive. For example, treatment of OPG and RANKL to vascular cells can either have no effect, promote, or inhibit vascular calcification *in vitro* [6], [11], [12], [13], [14], [15], [16]. *In vivo*, RANKL^−/−^ mice develop severe osteopetrosis and have no osteoclasts due to a lack of osteoclastogenesis [17] and recent studies using transgenic mice overexpressing RANKL in vascular smooth muscle cells (VSMCs) however, demonstrated enhanced vascular mineralisation potential [18]. The RANKL neutralizing antibody Denosumab, is currently used to treat osteoporosis; studies show that it may also inhibit vascular calcification *in vivo* [11]. This suggests the possible involvement of RANKL in vascular calcification. Contrarily, OPG-deficient mice develop osteoporosis, but also develop vascular calcification of great arteries (aorta, renal arteries), suggesting a protective role for OPG in the vasculature [19]. Moreover, in cardiovascular diseases and diabetes, RANKL is positively associated with circulating OPG [20], [21], [22], [23]. We recently demonstrated that OPG can inhibit calcification of VSMCs *in vitro* and that OPG accumulates in calcified human atherosclerotic tissue [16]. OPG is also a soluble receptor for TNF-related apoptosis-inducing ligand (TRAIL) [24].

TRAIL is a type II transmembrane protein of the TNF family of ligands that can also be cleaved to produce a soluble form. TRAIL was discovered in 1995 for its ability to induce apoptosis in cancer cells, without affecting other normal cells [25]. In humans, TRAIL initiates apoptosis by binding its death-domain containing receptors DR4 and DR5. Decoy receptors for TRAIL, either lacking a death-domain (decoy-receptor-1; DcR1) or containing a partial death-domain (decoy-receptor-2; DcR2), have also been identified. Decoy receptors have the ability to compete with death-receptor binding, and subsequently inhibit the induction of apoptosis; these include not only DcR1 and DcR2, but also OPG. In mice however, only one death-receptor has been identified, mDR5. Decoy receptors for TRAIL in mice have also been recognised, namely mDcR1, mDcR2 (consisting of 2 isoforms; membrane-bound mDcR2 and the secreted mDcR2) and OPG (reviewed in [24]). There is now growing evidence demonstrating pleiotropic functions for TRAIL *in vitro* and *in vivo;* this is not surprising since non-apoptotic signaling regulating cell survival, proliferation, migration and differentiation, have also been reported upon TRAIL-receptor activation [24], [26], [27], [28].

We recently showed that TRAIL is protective against atherosclerosis, since a deficiency of TRAIL in ApoE^−/−^ mice in response to a high fat diet (HFD) for 12 w, significantly increased arterial plaque area compared to ApoE^−/−^ mice [29]. While it is unclear as to whether TRAIL modulates calcification in the vasculature, multiple studies suggest that TRAIL may play a role in this pathophysiological process. For example, TRAIL and OPG expression is spatially distributed in lesions of Mönckeberg’s sclerosis, adjacent to vascular calcification [30], and TRAIL expression has been observed in calcified areas of abdominal aortic aneurysms [31]. In support of these, phosphate-induced mineralisation of human VSMCs was stimulated by TRAIL *in vitro* [32] and soluble TRAIL levels in sera of hemodialysis patients, a high risk group for cardiovascular diseases, are reduced [32]. The precise role of TRAIL *in vivo* in vascular calcification however is unclear. The aim of the present study is to understand the link between TRAIL, OPG, RANKL and vascular calcification *in vitro,* and *in vivo* in atherosclerotic lesions of TRAIL^−/−^ApoE^−/−^ mice.

## Methods

### Ethics statement

All animals were handled according to the Animal Care and Ethics Committee (ACEC) guidelines at UNSW (Sydney, Australia); the protocol was approved by the ACEC of UNSW (Ethics approval number 11/71B). To minimise stress, mice were monitored and handled daily.

### Primary cell Isolation and mice

Primary mouse TRAIL^−/−^ and wild-type C57Bl6 (WT) VSMCs were isolated from whole aortas as previously described [33]. Six week old male TRAIL^−/−^ApoE^−/−^ and ApoE^−/−^ mice [29] weighing approximately 16–18 g were placed on a HFD (Semi-Pure Rodent Diet, SF00-219, Specialty Feeds) for 12 and 20 w in specific pathogen-free conditions. At the end of the diet, mice were culled by cardiac exsanguination. Brachiocephalic arteries and aortas were excised, fixed in 10% formaldehyde for immunohistochemistry or snap frozen for expression studies. Plasma obtained at time of sacrifice was used for subsequent analysis of soluble OPG (R&D Systems) and RANKL (Merck Millipore).

### Cell culture

Primary human aortic VSMCs purchased from ATCC, were cultured in Waymouth’s medium (Invitrogen). TRAIL^−/−^ and WT VSMCs were cultured in high glucose DMEM medium (Sigma Aldrich). Where calcium was added exogenously, cells were serum-starved in M199 (Sigma-Aldrich) containing 1.36 mM calcium for 24 hours, prior to additional calcium at the indicated final concentrations and times. All media were supplemented with 10 or 20% fetal bovine serum (FBS), 10 µg/ml streptomycin, 10 U/ml penicillin and 1 mM L-glutamine. Cells were maintained at 37°C in a humidified atmosphere of 5% CO_2._ Human cells were not used beyond passage 10, and mouse cells were not used beyond passage 11.

### In vitro calcification assay

Prior to the addition of exogenous calcium, human VSMCs were serum-starved in M199 at approximately 90% confluence for 24 h. Total calcium (3.3 mM) was added every 2–3 days with fresh media, together with recombinant human TRAIL (1 and 10 ng/ml; R&D Systems); cells were harvested at day 10. For calcification studies involving murine cells, VSMCs were serum-starved for 24 h at 90% confluence. Calcium was added the following day with fresh media; Cells were harvested 2 days later. Quantification of calcification was performed by Alizarin red staining as previously described [16].

### Histology and immunochemistry

Haemotoxylin and eosin was used to assess tissue architecture of brachiocephalic arteries. Arteries were stained for Alizarin red and collagen II (1:100; Abcam). Digital images of arteries were captured using an Olympus DP72 microscope (Olympus) and quantification of indicated stains were performed as previously described [29]. Briefly, the percentage of positive staining in the plaque of brachiocephalic arteries/mouse was analysed. The threshold for the positive stain was determined and the sections were analysed by an investigator blinded to the genotype of the mice.

### RNA extraction, cDNA synthesis and quantitative PCR (qPCR)

Aortas were snap frozen in liquid nitrogen at time of sacrifice and stored at –80°C until further use. RNA was extracted from tissue using the RNeasy fibrous tissue kit from Qiagen. WT VSMCs were seeded into 6 well titre plates. At 80% confluence, the cells were serum arrested in M199 for 24 h followed by extraction of RNA using TriReagent (Sigma) [33]. RNA was extracted using TriReagent as above. cDNA was generated using iSCRIPT (Bio-Rad). qPCR was performed using the Rotor-Gene 6000 (Corbett Research) and SensiFast (Bioline) in triplicate. Relative changes in gene expression between groups was determined using the 2-▵▵cT^[29]^ method and values were normalised to levels of β-actin, which did not significantly differ. Where calcium was added exogenously, 18S RNA was used as a house keeper as calcium significantly altered the expression of β-actin. The list of primers for each gene can be found in Table 1.

**Table 1:** Murine primer sequences.

Mouse Primers		
Gene	Forward Primer 5′- 3′	Reverse Primer 5′- 3′
**IL-1β**	GTTTCTGCTTTCACCACTCCA	GAGTCCAATTTACTCCAGGTCAG
**TNF-α**	CAGGCGGTGCCTATGTCTC	CGATCACCCCGAAGTTCAGTAG
**PPAR-γ**	CACAATGCCATCAGGTTTGG	GCTGGTCGATATCACTGGAGATC
**OPG**	ATCAGAGCCTCATCACCTT	CTTAGGTCCAACTACAGAGGAAC
**RANKL**	CCAGCTATGATGGAAGGCTCA	ACCGAAGATAATGGACATGC
**Collagen II**	GAAGGTGGAAAGCAAGGTGA	CATCAGTACCAGGAGTGCCA
**BMP-2**	GGGACCCGCTGTCTTCTAGT	TCAACTCAAATTCGCTGAGGAC
**Runx2**	AACGATCTGAGATTTGTGGGC	CCTGCGTGGGATTTCTTGGTT
**β-actin**	AACCGTGAAAAGATGACCCAGAT	CACAGCCTGGATGGCTACGTA
**18S RNA**	CGGCTACCACATCCAAGGAA	GCTGGAATTACCGCGGCT

### Statistics

GraphPad Prism version 5.0 (GraphPad Software, San Diego, CA, USA) was used to analyse data with results expressed as mean ± SEM. Statistical comparisons were performed where appropriate, using either a Student’s *t* test, or one or two way ANOVA (with Bonferroni’s multiple comparison test) where appropriate. *p*<0.05 was considered significant.

## Results

### TRAIL is protective of calcium-induced VSMC calcification *in vitro*

**Figure 1:**
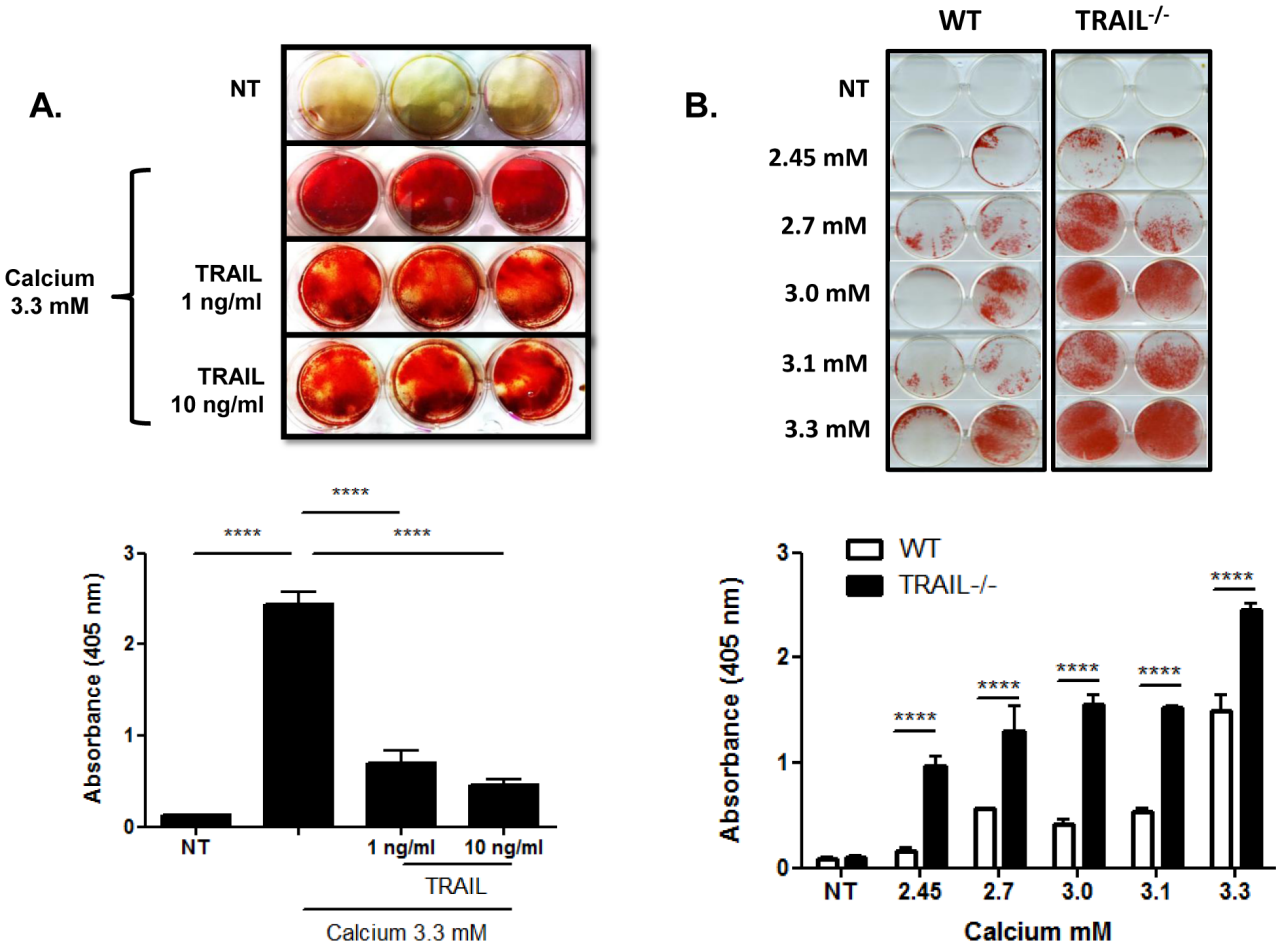
Calcium-induced calcification is blocked by TRAIL in VSMCs. (**A**) Human VSMCs were treated with TRAIL and calcium (3.3 mM) every 2–3 d. Cells were serum-starved for 24 h prior to treatment for 10 d. Representative photograph of each treatment group. Alizarin red staining indicates calcification. Cells were fixed and calcification assessed using an alizarin red-based assay. (**B**) WT and TRAIL^−/−^ VSMCs were treated with increasing concentrations of calcium (2.45–3.3 mM). Cells were serum-starved for 24 h prior to treatment for 2 d. Representative photograph of each treatment group. Alizarin red staining indicates calcification. Cells were fixed and calcification assessed using an alizarin red-based assay as described in the methods. NT, no treatment. ANOVA (n = 3 experiments); ****p<0.0001.

We recently demonstrated that addition of soluble OPG to human VSMCs can inhibit calcium-induced calcification *in vitro* [16]. Since OPG is a soluble receptor for TRAIL, we next investigated whether TRAIL could also influence calcium-induced calcification in these cells. Human VSMCs were exposed to recombinant TRAIL, and an *in vitro* alizarin red-based assay [16], [34] was used to assess calcification stimulated by exogenous supraphysiological calcium concentrations for 10 days. No calcification was observed in untreated cells exposed to 1.36 mM calcium already present in M199 medium (Fig. 1A). In contrast, cells exposed to a total calcium concentration of 3.3 mM, displayed significant increases in alizarin red staining, demonstrating calcification of human VSMCs (Fig. 1A). Interestingly, VSMCs exposed to calcium and TRAIL, at both 1 and 10 ng/ml for 10 days significantly inhibited calcium-induced calcification (Fig. 1A). In support of a protective role for TRAIL in calcium-induced calcification of VSMCs, murine TRAIL^−/−^ VSMCs exhibited accelerated calcification within 2 days of treatment, and in a dose-dependent manner (Fig. 1B). Interestingly, in these cells, calcification was observed even at the lowest calcium concentration of 2.45 mM (Fig. 1B). Taken together, these findings highlight the importance of TRAIL in protecting against calcium-induced calcification of VSMCs *in vitro*.

### Calcium differentially regulates RANKL, OPG and TRAIL

**Figure 2:**
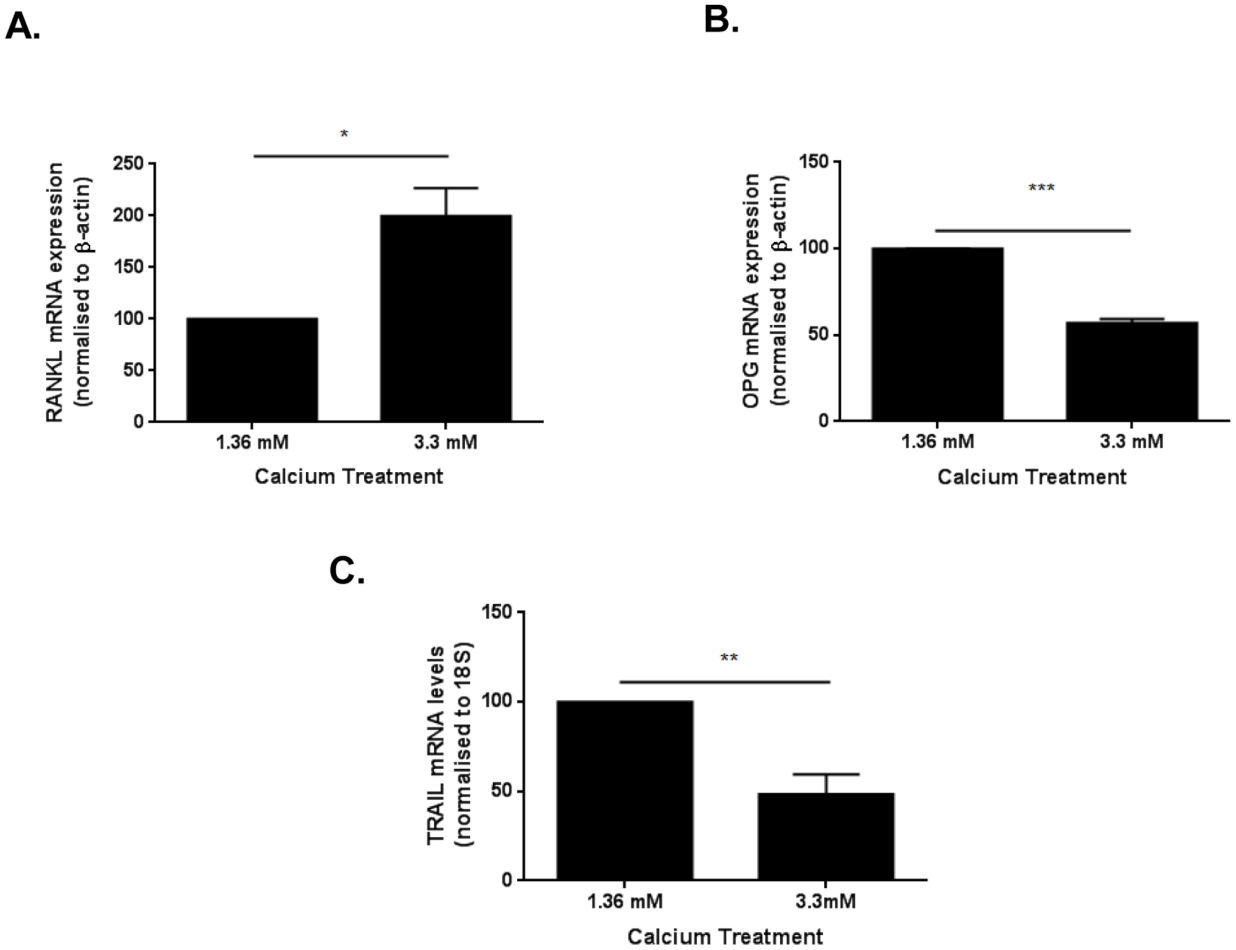
Calcium differentially regulates RANKL, OPG and TRAIL expression in VSMCs. Relative changes in mRNA levels of (**A**) RANKL, (**B**) OPG and (**C**) TRAIL in WT VSMCs treated with calcium using the 2ΔΔCt method. WT cells were serum-starved for 24 h prior to treatment with total 3.3 mM calcium. RNA was extracted 24 h later and real-time PCR was performed in triplicate. Expression was normalised to 18S RNA. Changes in mRNA expression were compared with untreated WT expression. Mann-Whitney t-test (n = 3 experiments); *p<0.05; **p<0.01.

The OPG and RANKL cytokine system regulate bone metabolism, they are also implicated in vascular calcification [8]. We have recently shown that OPG can inhibit calcium-induced calcification of human VSMCs, and OPG itself is regulated by calcium *in vitro* [16]. We next wanted to assess whether calcium could also regulate the expression of RANKL and TRAIL. Calcium treatment for 24 h significantly increased RANKL expression in WT VSMCs (Fig 2A). In contrast, calcium significantly inhibited OPG and TRAIL mRNA (Fig. 2B-C), almost a mirror image of RANKL. These studies demonstrate that calcium at 3.3 mM differentially regulates the expression of RANKL, OPG and TRAIL at 24 h exposure to modulate calcium-induced vascular calcification of VSMCs *in vitro*.

### Arteries from HFD fed TRAIL^−/−^ApoE^−/−^ mice display increased cartilaginous metaplasia and calcification

**Figure 3:**
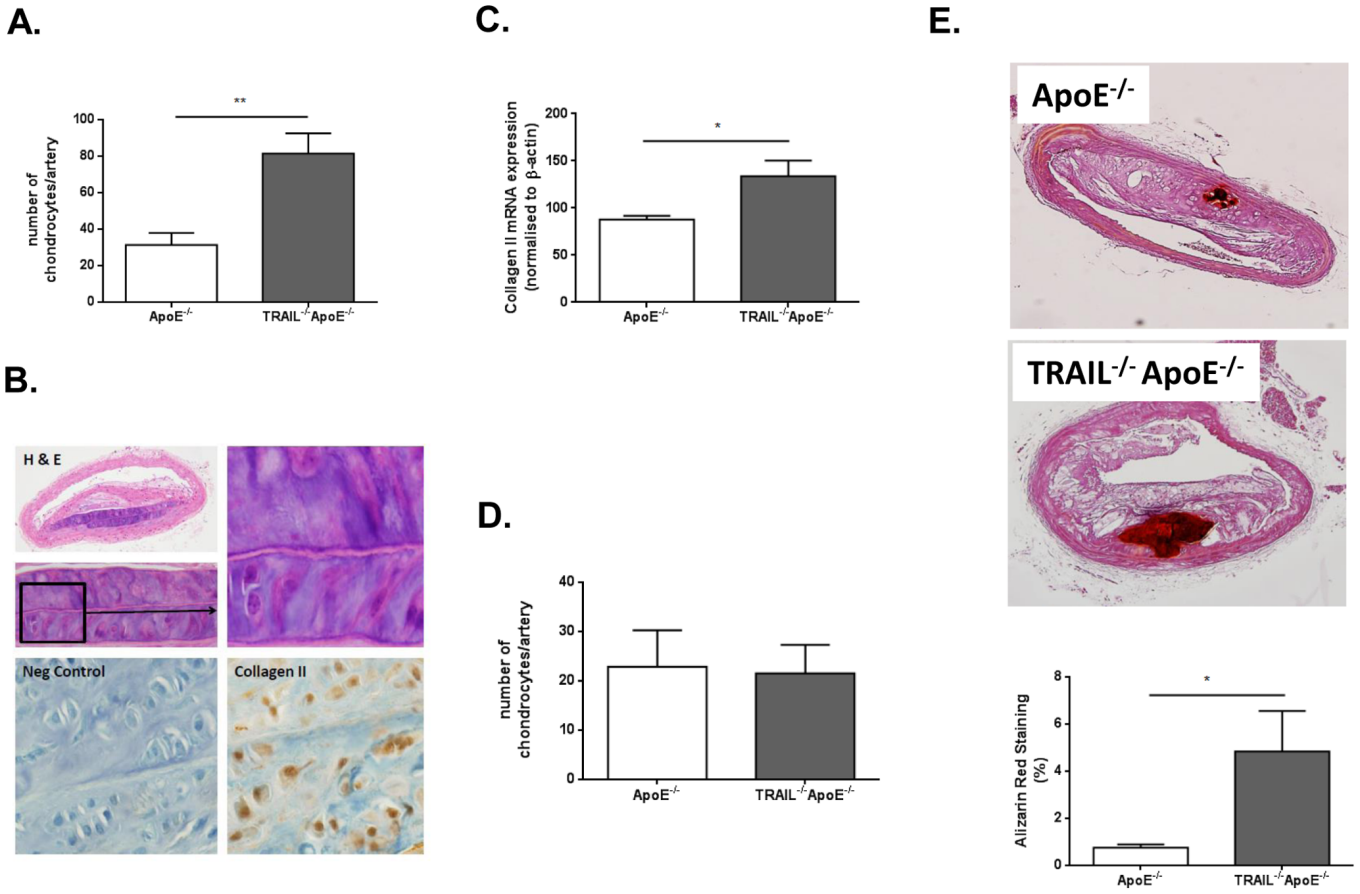
TRAIL-deficiency promotes vascular cartilaginous metaplasia and calcification. (**A**) Quantification of chondrocyte-like cells from ApoE^−/−^ and TRAIL^−/−^ApoE^−/−^ mice fed a HFD for 12 w. Mann-Whitney t-test (n = 8); **p<0.01. (**B**) Representative cross section of TRAIL^−/−^ApoE^−/−^ brachiocephalic arteries stained for H&E and collagen II (20x magnification). (**C**) Collagen II mRNA expression from aortas of 12 w HFD TRAIL^−/−^ApoE^−/−^ and ApoE^−/−^ mice (n = 4-6/genotype). (**D**) Quantification of chondrocyte-like cells from ApoE^−/−^ and TRAIL^−/−^ApoE^−/−^ mice fed a HFD for 20 w. Mann-Whitney t-test (n = 6-8/genotype). (**E**) Representative cross section of ApoE^−/−^ and TRAIL^−/−^ApoE^−/−^ brachiocephalic arteries stained for alizarin red (10x magnification) and quantification of the staining. Mann-Whitney t-test (n = 6-8/genotype) *p<0.05.

In the vasculature, it is suggested that similar mechanisms for cartilage and bone formation occur as ectopic calcification, and in fact, cells and proteins from bone tissue can be found in the vascular wall [35]. Chondrocyte-like cells and calcification have been observed within the fibro-fatty plaque of brachiocephalic arteries from 60 w chow fed ApoE^−/−^ mice [36]. We have previously shown that TRAIL^−/−^ApoE^−/−^ mice on a HFD for 12 w resulted in accelerated atherosclerosis when compared to ApoE^−/−^ alone [29]. To examine the effect of TRAIL on calcification in advanced atherosclerotic lesions, TRAIL^−/−^ApoE^−/−^ and ApoE^−/−^ mice were placed on a HFD for 12 and 20 w. Brachiocephalic arteries are among the most commonly studied vessels in the analysis of atherosclerosis in murine models, and examination of those from TRAIL^−/−^ApoE^−/−^ and ApoE^−/−^ mice on 12 w HFD exhibited significant changes in cellular composition, particularly with the detection of chondrocyte-like cells and cartilaginous metaplasia. Compared to ApoE^−/−^, significant increases in chondrocyte-like cell number were observed in arteries of 12 w HFD TRAIL^−/−^ApoE^−/−^ mice (Fig. 3A). The intermediate stage of chondrocyte differentiation and cartilage production in bone is evident by collagen II expression [37]. Specific collagen II staining was apparent in 12 w HFD TRAIL^−/−^ApoE^−/−^ arteries with chondrocytes (Fig. 3B). Consistent with this, aortas of 12 w HFD-fed TRAIL^−/−^ApoE^−/−^ mice had significantly elevated collagen II mRNA expression (Fig 3C). By 20 w however, no changes in chondrocyte numbers were seen in plaque between genotypes (Fig. 3D), nor was there any change in aortic collagen II expression (data not shown). Importantly, compared to controls, 20 w HFD TRAIL^−/−^ApoE^−/−^ arteries displayed significantly increased calcification as assessed by alizarin red staining (Fig. 3E), a finding not observed at 12 w (data not shown). Taken together, these data suggest that TRAIL-deficiency accelerates chondrocyte development and calcification in atherosclerosis *in vivo*.

### Aortas of TRAIL^−/−^ApoE^−/−^ mice have increased expression of calcification-regulating genes and inflammatory cytokines

Since calcium deposits commonly occur in the greater vessels [38], we next examined the expression of OPG and RANKL from the aortas of HFD TRAIL^−/−^ApoE^−/−^ and ApoE^−/−^ mice. No change in cellular OPG mRNA was observed in aortas of 12 and 20 w HFD-fed animals (Tables 2 and 3). In contrast, RANKL expression was significantly increased in aortas of 12 w HFD fed TRAIL^−/−^ApoE^−/−^ (Table 2). Bone morphogenic proteins (BMP) e.g. BMP-2 and the master osteoblast transcription factor Runx2, are important in the development of cartilage and bone [39], [40]. Of note, BMP-2 can regulate Runx2 [41], and both factors can regulate RANKL [42], [43]. While BMP-2 expression was significantly elevated from 12 w HFD TRAIL^−/−^ApoE^−/−^ aortas, no change in Runx2 expression was observed between genotype (Table 2). Like RANKL, BMP-2 and Runx2 expression did not differ between genotype at 20 w (Table 3).

**Table 2:** Aortas from 12 w HFD-fed TRAIL^−/−^ApoE^−/−^ mice display altered expression of bone markers and inflammation.

mRNA(relative to β-actin)	12 w	
	ApoE^−/−^	TRAIL^−/−^ApoE^−/−^
***OPG***	100±16.0	114.8±7.8
***RANKL***	100±6.5	144.2±19.9*
***BMP-2***	100±7.8	122.8±2.9*
***Runx2***	100±15.9	62.2±9.7
***IL-1β***	100±12.7	291.1±50.6*
***PPARγ***	100±6.0	243.3±11.5**
***TNFα***	100±17.9	131±3.9

ANOVA (n = 4-6/genotype); *p<0.05, **p<0.01.

**Table 3:** Aortas from 20 w TRAIL^−/−^ApoE^−/−^ mice display increased inflammation.

mRNA(relative to β-actin)	20 w	
	ApoE^−/−^	TRAIL^−/−^ApoE^−/−^
***OPG***	100±27.3	113.9±21.1
***RANKL***	100±25.0	122.2±19.2
***BMP-2***	100±11.1	103.2±10.88
***Runx2***	100±12.9	88.9±4.3
***IL-1β***	100±10.9	212.9±45.8*
***PPARγ***	100±23.5	114.9±26.9
***TNFα***	100±31.4	114.5±28.7

ANOVA (n = 5/genotype); *p<0.05.

Inflammatory markers such as IL-1β, PPARγ and TNF-α are implicated in bone turnover [44], [45]. Therefore, we next assessed the expression of these markers in diseased aortas of 12 and 20 w HFD-fed TRAIL^−/−^ApoE^−/−^ and ApoE^−/−^ mice. IL-1β and PPARγ mRNA expression was significantly increased in TRAIL^−/−^ApoE^−/−^ aortas at 12 w HFD (Table 2). While a trend for increased TNF-α expression was observed in 12 w TRAIL-deficient aortas, this increase was not significant (Table 2). Interestingly in TRAIL^−/−^ApoE^−/−^ arteries where calcification was already evident (20 w), increases in aortic mRNA expression of RANKL, PPARγ and to some extent TNF-α were no longer observed (Table 3). In contrast, IL-1β expression was still significantly elevated at 20 w (Table 3). When we examined circulating levels of OPG and RANKL, OPG levels were slightly elevated and reaching significance at 12 w in TRAIL^−/−^ApoE^−/−^ mice, with no significant changes observed by 20 w (Table 4). No differences in plasma RANKL were observed (Table 4). These findings provide further evidence that TRAIL deficiency results in increased expression of cellular RANKL, chondrogenic bone markers and inflammatory mediators, which in part may lead to accelerated cartilage/bone formation and calcification in the vessel wall.

**Table 4:** Circulating OPG and RANKL levels.

	12 w		20 w	
*Fold Change*	ApoE^−/−^	TRAIL^−/−^ApoE^−/−^	ApoE^−/−^	TRAIL^−/−^ApoE^−/−^
***OPG***	1.00±0.06	1.4±0.08**	1.00±0.04	0.85±0.12
***RANKL***	1.00±0.15	1.05±0.10	1.00±0.12	0.78±0.13

Plasma OPG levels (ELISA) and plasma RANKL (Milliplex assay) levels were measured from ApoE^−/−^ and TRAIL^−/−^ApoE^−/−^ mice 12 and 20 w of HFD. Fold change compared to control ApoE^−/−^ mice. ANOVA (n = 6-8/genotype); **p<0.01.

## Discussion

**Figure 4:**
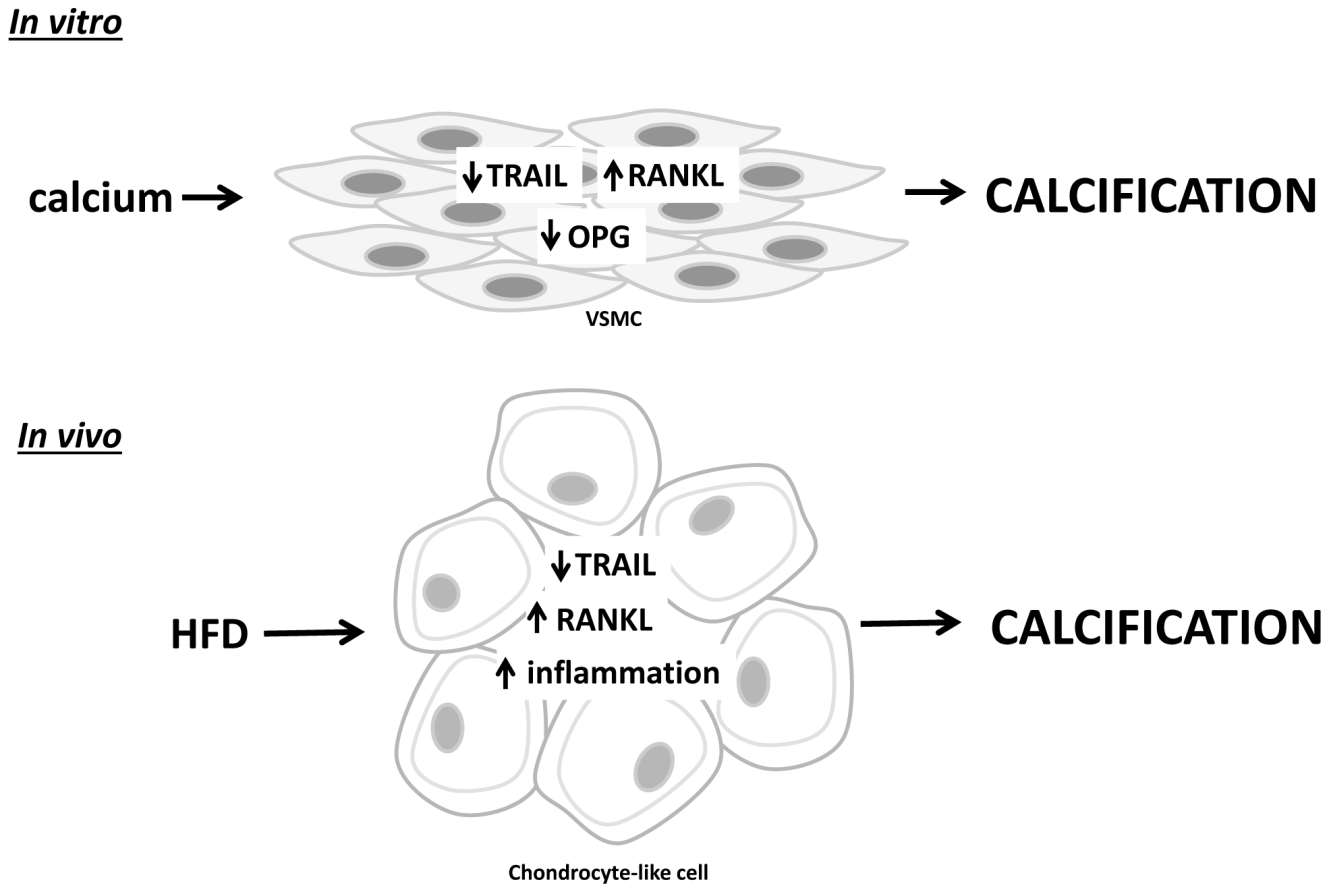
Model for the protective action of TRAIL in vascular calcification. Calcium differentially regulates RANKL, OPG and TRAIL expression in VSMCs. As a consequence, a deficiency in TRAIL with the addition of exogenous calcium leads to vascular calcification *in vitro*. *In vivo*, TRAIL-deficiency in ApoE^−/−^ mice on a HFD increases the expression of cellular RANKL and inflammatory cytokines within the vessel wall, simultaneously leading to an acceleration of cartilage development and subsequent calcification.

Two cytokines that are responsible for controlling osteoclast biology are RANKL and OPG. RANKL binds to its cellular receptor RANK to initiate osteoclastogenesis [8], [9]. In contrast, binding of RANKL to its decoy receptor OPG, results in inhibition of osteoclastogenesis [10]. While the OPG/RANKL cytokine system has been extensively studied in bone metabolism, animal models suggest that this system is also implicated in controlling vascular calcification [46], [47], [48]. In the present study we demonstrate for the first time that TRAIL modulates RANKL, such that TRAIL deficiency in the vessel wall of ApoE^−/−^ mice fed a HFD, results in increased cellular RANKL expression, which leads to vascular calcification (Fig. 4). These findings are similar to those in ApoE^−/−^mice after ovariectomy, which exhibit atherosclerotic calcification, osteoporosis and increased expression of RANKL [49].

Chondrocyte-like cells promoting cartilage formation are implicated in calcification in human atherosclerosis [3]. Here we show that RANKL’s expression and role in controlling bone turn-over in the vessel wall involves TRAIL. Interestingly, RANKL’s increase in expression in diseased TRAIL^−/−^ApoE^−/−^ aortas was time-dependent; increased at 12 w in TRAIL^−/−^ApoE^−/−^ mice displaying increased arterial cartilage, with no significant changes in RANKL expression between genotypes by 20 w. A possible stimulus for RANKL expression at 12 w in TRAIL^−/−^ApoE^−/−^ mice may be BMP-2, an important anabolic factor in bone formation and mineralization [39]. During bone formation, BMP-2-induced osteoblastic differentiation in mesenchymal cells involves BMP-2-induced Runx2 expression [41]. Both BMP-2 and Runx2 can regulate RANKL expression [42], [43]. While we did not see increased Runx2 expression in TRAIL-deficient aortas, BMP-2 expression was significantly elevated at 12 w. Intriguingly, RANKL can directly regulate BMP-2 [50], suggesting a possible feedback mechanism in our system. These findings therefore, raise further questions on TRAIL-dependent calcification effects. Further investigations are needed to fully elucidate TRAIL’s role in these complex processes.

In addition to inflammation, many cytokines can act to promote bone resorption. For example, both IL-1β and TNFα can induce the expression of genes regulating cartilage resulting in degradation of matrix components [51]. Here we show that cellular mRNA levels of IL-1β, PPARγ and to some extent TNFα were also simultaneously elevated in 12 w TRAIL^−/−^ApoE^−/−^ aortas. We have previously shown that TRAIL-deficiency in ApoE^−/−^ mice leads to increased CD11b +ve splenic leukocytes, elevated levels of inflammatory markers such as MCP-1, and significantly increased macrophage infiltration in their atherosclerotic tissue, suggesting that TRAIL may attenuate monocyte recruitment in areas of chronic inflammation [29]. On the other hand, RANKL can promote the recruitment and infiltration of monocytes/macrophages [52], known to stimulate VSMC calcification probably via increased secretion of inflammatory cytokines. Both TNFα and IL-1β can stimulate the release of OPG from vascular cells, as well as induce RANKL expression [53]. This is consistent with the observation that TRAIL^−/−^ApoE^−/−^ mice displayed increased circulating OPG, with no change in cellular OPG levels at 12 w. This finding is consistent with a variety of clinical studies demonstrating increases in circulating OPG correlating with vascular disease [54], [55], [56]. The significance of circulating OPG levels is not fully established. For example, it is not clear if levels of circulating OPG are directly involved in promoting vascular calcification, or whether increased levels of OPG may reflect attempts to block excessive mineralisation in calcified atherosclerotic tissue [57].

PPARγ which is expressed by VSMCs in atherosclerotic lesions can also be induced by IL-1β and TNFα, in the same manner that they stimulate OPG. Interestingly, PPARγ ligands have been shown to restrain OPG levels generated by inflammatory cytokines suggesting a possible negative feedback mechanism [58]. This may provide a potential mechanism for the normalised levels of OPG observed in TRAIL-deficient animals by 20 w. Our findings suggest that RANKL may be an inducer or a marker of cartilage/bone formation in the vasculature. This is corroborated by the *in vitro* finding that insulin-mediated osteoblastic differentiation of VSMCs is promoted by increased RANKL expression [59]. Furthermore, osteoclast differentiation induced by RANKL was inhibited by TRAIL [60]. Of note, TRAIL also induces chondrocyte apoptosis [61]. These studies suggest that TRAIL may be an important player in the regulation of the OPG/RANKL cytokine system.

TRAIL has been shown to regulate VSMC proliferation and neointimal thickening after injury [33], [62], [63], as well as in a rodent model of pulmonary arterial hypertension [64]. While these studies suggest that TRAIL can promote atherogenesis and vascular disease, serum concentrations in patients with coronary artery disease (CAD) have demonstrated lower TRAIL levels inversely associated with the severity of CAD, and especially lower levels in patients with acute coronary syndromes, suggesting a protective role in cardiovascular diseases [44], [52]. Moreover, low circulating TRAIL levels are associated with all-cause and cardiovascular mortality [19], and consistent with this, rodent models of atherosclerosis also suggest a protective role for TRAIL against atherosclerosis [29], [65]. These findings suggest that TRAIL has differential roles at different stages of disease. These may be dependent on multiple factors including ligand concentration, cell types involved, and expression of its receptors. For example, in VSMCs, TRAIL-inducible proliferation is dependent on DR4 and DcR1 [63], [64], while T-cell mediated TRAIL-dependent apoptosis requires DR5 [66]. Interestingly, TRAIL protein is detected in stable atherosclerotic lesions, in vulnerable plaques, and also localized to the medial layer of arteries in Mönckeberg's sclerosis [52], [63]. Moreover, while TRAIL is expressed in regions of calcified vascular tissue [30], [31], circulating TRAIL levels in sera of hemodialysis patients which are prone to severe vascular calcification are reduced [32]. Interestingly, ApoE^−/−^ mice at 6 w prior to the start of their HFD had higher circulating TRAIL levels compared to 12 w HFD ApoE^−/−^ mice (data not shown), suggesting that the HFD may attenuate TRAIL levels in these mice. ApoE^−/−^ mice also had chondrocyte-like cells and demonstrated some arterial calcification by 20 w, however, a total TRAIL-deficiency in ApoE^−/−^ mice, accelerated this process. It is enticing to speculate that a Western lifestyle with increased consumption of high calorie foods may be in part, responsible for the reduced circulating TRAIL levels observed in humans with disease [44], [52]. Our study suggests that TRAIL protects against atherosclerosis to some extent, by attenuating calcification as demonstrated by our *in vivo* and *in vitro* data. In conclusion, our study is the first to show the importance of TRAIL in regulating RANKL expression necessary for vascular calcification. This is the first demonstration implicating a protective role for TRAIL against calcification in the vasculature *in vivo*.
