# Correction: TRAIL-Deficiency Accelerates Vascular Calcification in Atherosclerosis via Modulation of RANKL

**DOI:** 10.1371/annotation/9732ca63-7532-4b92-8d6e-65c0863f25aa

**Published:** 2013-09-25

**Authors:** Belinda A. Di Bartolo, Siân P. Cartland, Hanis H. Harith, Yuri V. Bobryshev, Michael Schoppet, Mary M. Kavurma

This article has been republished to correct an error in the listed Editor. 

